# Long‐term survival analysis of patients with stage IIIB‐IV non‐small cell lung cancer complicated by type 2 diabetes mellitus: A retrospective propensity score matching analysis

**DOI:** 10.1111/1759-7714.14676

**Published:** 2022-10-11

**Authors:** Xuejiao Li, Haiyan Fang, Dongwei Zhang, Liming Xia, Xiang Wang, Jingping Yang, Shaohu Zhang, Ya Su, Yongfu Zhu

**Affiliations:** ^1^ School of Nursing Anhui University of Traditional Chinese Medicine Hefei China; ^2^ The First Department of Oncology The First Affiliated Hospital of Anhui University of Traditional Chinese Medicine Hefei China

**Keywords:** non‐small cell lung cancer, diabetes mellitus, chemotherapy, survival

## Abstract

**Background:**

This study aimed to determine the effect of type 2 diabetes mellitus (T2DM) on overall survival (OS) of patients with stage IIIB–IV non‐small cell lung cancer (NSCLC).

**Methods:**

We retrospectively analyzed patients with stage IIIB–IV NSCLC from January 2015 to December 2018 in the Department of Oncology at the First Affiliated Hospital of Anhui University of Traditional Chinese Medicine. Kaplan–Meier plots, log‐rank tests, and Cox proportional hazards regression models were used to describe the effect of T2DM on the OS of patients with stage IIIB–IV NSCLC.

**Results:**

This study collected data on 76 patients with NSCLC and T2DM (group A) and 214 NSCLC patients without T2DM (group B). After propensity score matching (PSM) analysis, 74 patients were included in each group. The mean OS of all patients was 17 months (range, 11–31 months). The mean OS of group A was 15 months (range, 8–25 months) and the mean OS of group B was 20 months (range, 14–39 months). The mean OS of group B was longer than group A, and the difference was statistically significant. Univariate analysis of the clinical data showed that T2DM and complications were significantly correlated with the prognosis of patients with stage IIIB‐IV NSCLC (*p* = 0.003 and *p* = 0.034). Multivariate Cox model analysis showed that T2DM and complications were independent prognostic factors for patients with stage IIIB–IV NSCLC (*p* = 0.002 and *p* = 0.024, respectively).

**Conclusion:**

Stage IIIB–IV NSCLC patients without T2DM have an increased OS compared to patients with stage IIIB–IV NSCLE and T2DM.

## Graphical abstract

The mean OS of IIIB–IV NSCLC patients without T2DM was longer than IIIB–IV NSCLC patients with T2DM, and the difference was statistically significant.

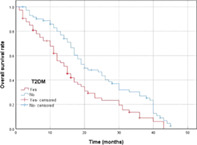

## INTRODUCTION

According to the latest cancer data released by the International Research Institute of the World Health Organization, lung cancer is the deadliest malignant tumor worldwide.^1^, ^2^ Lung cancer is the leading cause of morbidity and mortality amongst malignant tumors in China. Furthermore, 80% of the lung cancer cases in China are non‐small cell lung cancer (NSCLC) with a 5‐year survival rate < 20%.^3^, ^4^ As a result of an aging population and changes in lifestyle, an increasing number of lung cancer patients have other major diseases, such as diabetes mellitus (DM), hypertension, and cerebral infarction. Previous studies have shown that DM affects the prognosis of cancer patients, including patients with breast, colorectal, and liver cancers.^5^, ^6^, ^7^ In addition, it has been shown that type 2 DM (T2DM) promotes tumor cell proliferation and metastasis vis‐à‐vis hyperglycaemia and insulin‐like growth factor‐1(IGF‐1).^8^ In addition, it has been shown that DM‐induced modification of advanced glycation end‐products hinders invasive metastasis of lung cancer cells.^9^ Therefore, we reviewed the medical records of patients with NSCLC diagnosed in our hospital from January 2015 to December 2018 to analyze the clinicopathological characteristics of patients with T2DM, determine the effect of T2DM on the overall survival (OS) of patients with NSCLC, and evaluate whether T2DM affects the long‐term prognosis of patients with NSCLC.

## METHODS

### General information

This retrospective study was conducted with approval of the Institutional Review Board of the First Affiliated Hospital of Anhui University of Traditional Chinese Medicine. Data were collected from 76 patients with NSCLC and T2DM and 214 NSCLC patients without T2DM who were treated in the Department of Oncology of the First Affiliated Hospital at Anhui University of Traditional Chinese Medicine from January 2015 to December 2018. The patients were divided into two groups based on NSCLC that was or was not complicated by T2DM: group A with T2DM; and group B without T2DM. The case inclusion criteria were as follows: (1) histopathological confirmation of NSCLC, (2) pathological stage IIIB–IV, (3) treatment with first‐line platinum‐containing dual‐drug chemotherapy, (4) no history of other tumors, (5) no prior chemoradiotherapy, (6) performance status (PS) score of 0–1, and (7) T2DM diagnosed by an endocrinologist. The exclusion criteria were as follows: (1) NSCLC diagnosis preceding the diagnosis of T2DM, (2) a diagnosis of type 1 DM, (3) failure to complete first‐line chemotherapy, and (4) incomplete data. Group A met inclusion criteria 1–7 and group B met inclusion criteria 1–6. TNM staging was based on the eighth edition of the International Association for Lung Cancer Research (IASLC).

### Observation index

The clinicopathological data of the two groups of NSCLC patients were compared, the OS times of NSCLC patients with and without T2DM were compared, and the clinicopathological factors affecting the prognosis of NSCLC patients were analyzed.

### Chemotherapy regimen

All patients received paclitaxel plus cisplatin (TP regimen), docetaxel + cisplatin (DP regimen), or pemetrexed + cisplatin (PC regimen). Treatment efficacy was evaluated every two cycles. Patients who responded to treatment completed at least four cycles of chemotherapy, while nonresponsive patients were switched to other regimens or other treatments, including docetaxel, pemetrexed, and epidermal growth factor receptor tyrosine kinase inhibitors (EGFR‐TKIs).

### Prognostic follow‐up

Patient prognosis was determined by outpatient re‐examination, telephone contact, or social networking software. The patients were followed once monthly in the first 6 months and every 3 months thereafter. The follow‐up cutoff date was December 31, 2021, and the content of the follow‐up evaluation was related to OS, which refers to the time from the diagnosis of NSCLC to the date of death, the last follow‐up date, or the follow‐up deadline.

### Statistical analysis

The patient data (sex, age, smoking history, tumor location, pathological type, TNM staging, complications, performance status (PS) score, and chemotherapy regimen) were analyzed using SPSS 26.0 statistical software. The variables with the closest tendency score between the two groups were matched at a ratio of 1:1, and the clamp value was 0.02. Then, survival analysis was performed between the two groups. A *t*‐test was used to compare the mean of the measured data, and a χ2 test was used to compare the counted data. The survival rate was calculated using the Kaplan–Meier method, and the difference in survival rate between groups was analyzed using the log‐rank and trend tests. The Cox model was used for multivariate survival analysis. OS is expressed as P50 (P25 and P75). The difference was statistically significant at *p* < 0.05.

## RESULTS

### Clinicopathological characteristics of patients in the two groups

Before propensity score matching (PSM) analysis, there were no significant differences in sex, age, smoking history, tumor location, pathological type, TNM stage, PS score, or chemotherapy regimen between the two groups. The number of complications in group A was greater than group B (*p* < 0.001). After PSM analysis, 74 patients were included in each group. There were no significant differences in sex, age, smoking history, tumor location, pathological type, TNM stage, PS score, chemotherapy regimen, or complications between the two groups (Table 1).

**TABLE 1:** Characteristics of NSCLC patients by T2DM

Variable	PSM 前		χ2	*p*‐value	PSM 后		χ2	*p*‐ value
	A group (*n* = 76)	B group (*n* = 214)			A group (*n* = 74)	B group (*n* = 74)		
**Sex**			0.000	0.997			0.000	1.000
Male	54 (71.1%)	152 (71.0%)			52 (70.3%)	52 (70.3%)		
Female	22 (28.9%)	62(29.0%)			22 (29.7%)	22 (29.7%)		
**Age (year)**			0.285	0.594			0.029	0.866
≤ 60	30 (39.5%)	92 (43.0%)			28 (37.8%)	29 (39.2%)		
> 60	46 (60.5%)	122 (57.0%)			46 (62.2%)	45 (60.8%)		
**Smoking history**			0.083	0.773			0.142	0.707
Yes	20 (26.3%)	60 (28.0%)			20 (27.0%)	18 (24.3%)		
No	56 (73.7%)	154 (72.0%)			54 (73.0%)	56 (75.7%)		
**Tumor location**			2.129	0.712			2.157	0.707
RUL	14 (18.4%)	48 (22.4%)			14 (18.9%)	12 (16.2%)		
RML	4 (5.3%)	20 (9.3%)			4 (5.4%)	5 (6.8%)		
RLL	18 (23.7%)	46 (21.5%)			18 (24.3%)	16 (21.6%)		
LUL	24 (31.6%)	62 (29.0%)			24 (32.4%)	20 (27.0%)		
LLL	16 (21.1%)	38 (17.8%)			14 (18.9%)	21 (28.4%)		
**Pathological type**			5.253	0.072			1.817	0.403
Adenocarcinoma	42 (55.3%)	140 (65.4%)			42 (56.8%)	41 (55.4%)		
Squamous cell carcinoma	26 (34.2%)	66 (30.8%)			24 (32.4%)	29 (39.2%)		
Other	8 (10.5%)	8 (3.7%)			8 (10.8%)	4 (5.4%)		
**TNM stage**			0.005	0.944			0.108	0.742
IIIB stage	38 (50.0%)	106 (49.5%)			38 (51.4%)	40 (54.1%)		
IV stage	38 (50.0%)	108 (50.5%)			36 (48.6%)	34 (45.9%)		
**PS score**			2.143	0.143			0.035	0.852
0	20 (26.3%)	76 (35.5%)			20 (27.0%)	19 (25.7%)		
1	56 (73.7%)	138 (64.5%)			54 (73.0%)	55 (74.3%)		
**Chemotherapy regimen**			1.091	0.579			0.714	0.700
TP	10 (13.2%)	38 (17.8%)			10 (13.5%)	12 (16.2%)		
DP	16 (21.1%)	48 (22.4%)			16 (21.6%)	19 (14.9%)		
PC	50 (65.8%)	128 (59.8%)			48 (63.8%)	43 (13.5%)		
**Complications**			12.116	<0.001			0.000	1.000
Yes	32 (42.1%)	46 (21.5%)			30 (40.5%)	30 (40.5%)		
No	44 (57.9%)	168 (78.5%)			44 (59.5%)	44 (59.5%)		

Abbreviations: DP, docetaxel + cisplatin regimen; LLL, left lower lobe: LUL, left upper lobe; PC, pemetrexed + cisplatin regimen; PS, performance status; PSM, propensity score matching; RLL, right lower lobe; RML, right middle lobe; RUL, right upper lobe; T2DM, type 2 diabetes mellitus; TNM stage, tumor node metastasis stage; TP, paclitaxel plus cisplatin regimen.

### **Effect of T2DM on OS in patients with stage IIIB–IV** **NSCLC**

**FIGURE 1:**
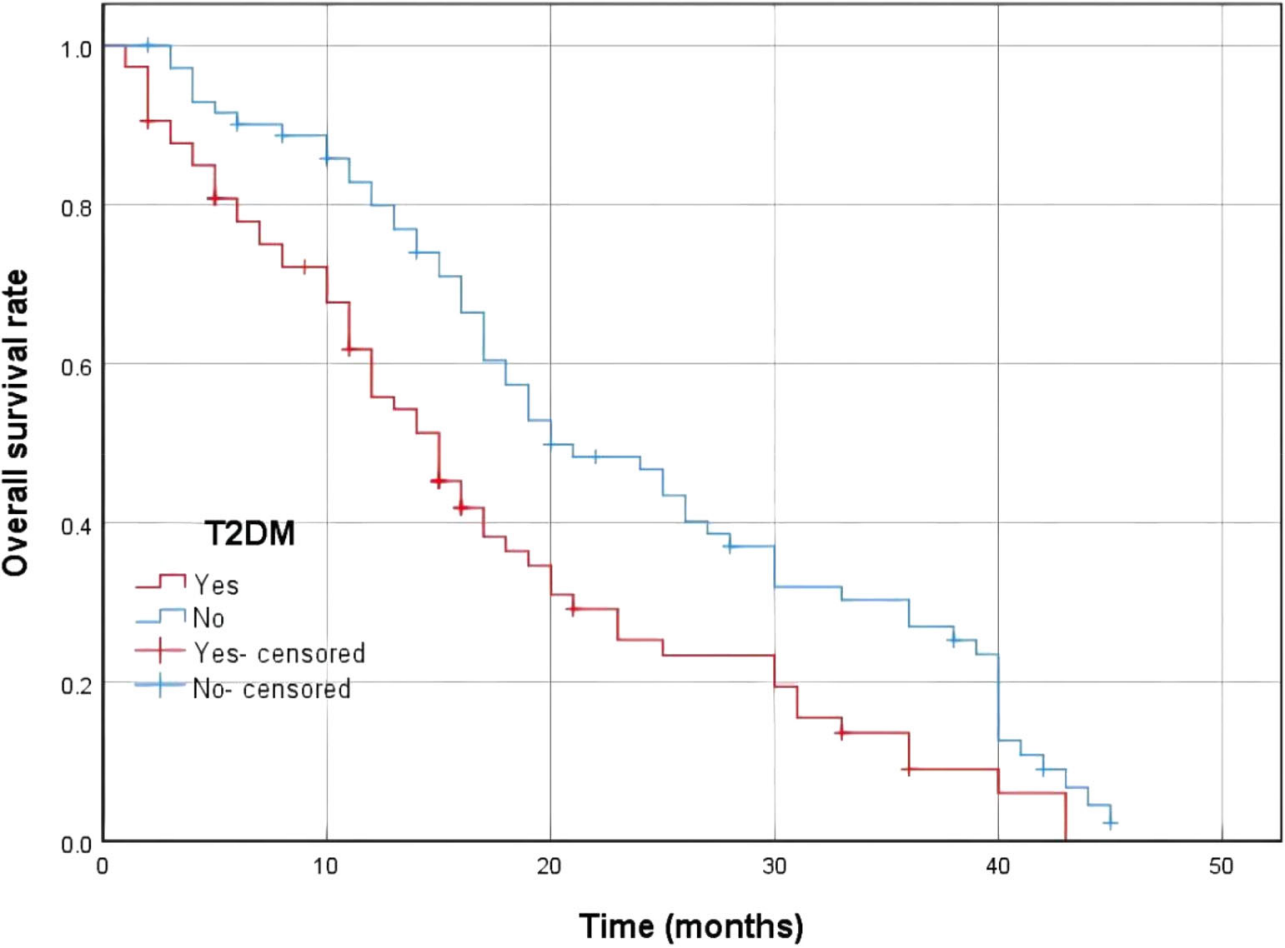
Kaplan‐Meier survival curves for A group and B group. (a) The median overall survival was significantly better in the B group. Group A with T2DM and Group B without T2DM

The mean OS of all patients was 17 months (range, 11–31 months). The mean OS of group A was 15 months (range, 8–25 months), and the mean OS of group B was 20 months (range, 14–39 months). The mean OS of group B was longer than group A; the difference was statistically significant (Figure 1).

### **Univariate and multivariate analyses of prognostic factors in patients with stage IIIB–IV** **NSCLC**

Univariate analysis of the clinical data showed that T2DM and complications were significantly correlated with the prognosis of patients with stage IIIB–IV NSCLC (*p* = 0.003 and *p* = 0.034, respectively; Table 2). Multivariate Cox model analysis showed that T2DM and complications were independent prognostic factors for patients with stage IIIB–IV NSCLC (*p* = 0.002 and *p* = 0.024, respectively; Table 3).

**TABLE 2:** Univariate analyses of prognostic factors and survival in patients with NSCLC

Variable	Case	Median survival time (month) (95% CI)	*p‐*value
**Sex**			0.784
Male	104	18	
Female	44	17	
**Age (year)**			0.706
≤60	57	17	
>60	91	17	
**Smoking history**			0.875
Yes	38	17	
No	110	17	
**Tumor location**			0.315
RUL	26	17	
RML	9	16	
RLL	34	20	
LUL	44	17	
LLL	35	19	
**Pathological type**			0.489
Adenocarcinoma	83	17	
Squamous cell carcinoma	53	20	
Other	12	15	
**TNM stage**			0.822
IIIB stage	78	17	
IV stage	70	20	
**PS score**			0.319
0	39	17	
1	109	18	
**Chemotherapy regimen**			0.528
TP	22	16	
DP	35	16	
PC	91	19	
**Complications**			0.034
Yes	60	13	
No	88	20	
**T2DM**			0.003
Yes	74	15	
No	74	20	

Abbreviations: DP, docetaxel + cisplatin regimen; LLL, left lower lobe: LUL, left upper lobe; PC, pemetrexed + cisplatin regimen; RLL, right lower lobe; RML, right middle lobe; RUL, right upper lobe; T2DM, type 2 diabetes mellitus; TNM stage, tumor node metastasis stage; TP, paclitaxel plus cisplatin regimen.

**TABLE 3:** Multivariate analyses of prognostic factors and survival in patients with non‐small cell lung cancer (NSCLC)

Variable	*β*	SE	Wald	HR	95% CI	*p*‐value
T2DM	0.306	0.098	9.773	1.357	(1.121, 1.644)	0.002
Complications	0.484	0.215	5.059	1.623	(1.064, 2.475)	0.024
Sex	−0.262	0.340	0.596	0.769	(0.395, 1.497)	0.440
Age	−0.156	0.209	0.555	0.856	(0.567, 1.290)	0.456
Smoking history	0.043	0.237	0.033	1.044	(0.656, 1.662)	0.856
Tumor location	0.088	0.071	1.528	1.092	(0.950, 1.255)	0.216
Pathological type	0.067	0.160	0.177	1.070	(0.782, 1.462)	0.674
TNM stage	−0.152	0.199	0.582	0.859	(0.582, 1.269)	0.446
PS score	−0.395	0.354	1.246	0.673	(0.336, 1.348)	0.264
Chemotherapy regimen	−0.001	0.079	0.000	0.999	(0.855, 1.167)	0.991

Abbreviations: HR, hazard ratio; LLL, left lower lobe: LUL, left upper lobe; RLL, right lower lobe; RML, right middle lobe; RUL, right upper lobe; SE, standard error; T2DM, type 2 diabetes mellitus; TNM stage, tumor node metastasis stage.

## DISCUSSION

As long ago as the 19th century, it was thought that DM affected the prognosis and OS of cancer patients.^10^ In recent years, related studies have reported that DM is a poor prognostic factor in patients with liver, gastric, and breast cancers.^5^, ^6^, ^7^ In addition, whether concomitant DM has an adverse effect on the prognosis of patients with NSCLC is controversial.^11^, ^12^, ^13^ Few studies have determined if T2DM has different outcomes on the prognosis of patients with stage IIIB–IV NSCLC. The current study showed that patients with stage IIIB–IV NSCLC and T2DM had a worse prognosis than stage IIIB–IV NSCLC patients without T2DM. In addition, T2DM is an independent factor affecting the prognosis of patients with stage IIIB–IV NSCLC.

Univariate and multivariate analyses showed that T2DM is a prognostic factor in patients with stage IIIB–IV NSCLC. Stage IIIB–IV NSCLC patients without T2DM had longer survival. A history of other chronic diseases is also a prognostic factor for patients with stage IIIB–IV NSCLC. Kirakli et al.^14^ studied the effect of the blood glucose level in 71 patients with stage IIIA–IIIB NSCLC on OS, disease‐free survival (DFS), and local recurrence after treatment, and showed that patients with hyperglycaemia and DM had a shorter survival time than patients with a normal blood glucose level. Diabetic patients had higher rates of relapse during treatment, and blood glucose levels were significantly higher in relapsed patients. Bergamino et al.^15^ retrospectively compared 56 advanced NSCLC patients with T2DM and 114 advanced NSCLC patients without T2DM and reported that the median progression‐free survival (PFS) and OS of patients with a fasting plasma glucose (FPG) ≥ 7 mmol/l was 8 and 15 months, respectively, and the median PFS and OS of patients with a FPG <7 mmol/l were 20 and 31 months, respectively. Thus, the fasting blood glucose level was an independent prognostic factor for advanced NSCLC.

The main pathological features of diabetic patients are hyperinsulinaemia and insulin resistance, and the concentration of insulin in the blood is increased.^16^ Insulin interferes with the synthesis of insulin‐like growth factor (IGF)‐binding protein in the liver, thereby increasing the level of free circulating IGF‐1 and promoting the IGF‐1 receptor (IGF‐1R) expression. Insulin and IGF‐1 are considered to be important factors in promoting the occurrence and development of tumors, and the activation of insulin receptors and IGF‐1R promote cell mitosis, inhibit cell apoptosis, and induce neovascularization.^17^, ^18^ Adequate energy supply is the premise for cancer cells to proliferate, invade, and metastasize rapidly and indefinitely. Tumors are preferentially powered by glycolysis. The higher the degree of tumor malignancy, the greater the energy demand and the more active glycolysis is. In addition, tumor cells are still preferentially powered by glycolysis under aerobic conditions, thus the “Warburg” effect.^19^ Hyperglycaemia not only provides sufficient energy for tumor cell proliferation to drive anabolism and cell division, but also a large number of intermediates produced in the process of glycolysis can provide raw materials for the synthesis of tumor organelles, so it is also conducive to tumor cell proliferation. In addition, the blood glucose of diabetic patients is more unstable, and tumor cells grow faster and are more likely to shed and metastasize when the blood glucose concentration fluctuates. Kim et al.^20^ showed that keratoblastoma pleomorphic lesions gradually increase without cell shedding when there is a continuous supply of glucose maintained at a specific concentration level, and the tumor lesions grow faster and tumor cells shed and metastasize when the glucose concentration fluctuates due to an intermittent supply of glucose.

This study has the following limitations. First, it was a single‐center retrospective analysis with a small sample size, and there was case selection bias. Thus, the results need to be confirmed by a large‐sample prospective study. Second, this study did not classify the hypoglycaemic drugs used by the patients and the statistics on the doses used, which may have led to bias in the results. Third, the impact of distant metastases on the prognosis of patients with stage IIIB–IV NSCLC was not analyzed.

In conclusion, concomitant T2DM is an independent factor that can affect the prognosis of patients with stage IIIB–IV NSCLC. Therefore, we should pay more attention to patients with stage IIIB–IV NSCLC with T2DM who are treated with chemotherapy.

## CONFLICT OF INTEREST

No authors report any conflict of interest.
